# Flavonoids improve type 2 diabetes mellitus and its complications: a review

**DOI:** 10.3389/fnut.2023.1192131

**Published:** 2023-05-31

**Authors:** Xinrui Yi, Mosi Dong, Naifei Guo, Jinlong Tian, Ping Lei, Song Wang, Yufeng Yang, Yan Shi

**Affiliations:** ^1^College of Traditional Chinese Medicine, Liaoning University of Traditional Chinese Medicine, Shenyang, China; ^2^Food Science College, Shenyang Agricultural University, Shenyang, China; ^3^Liaoning Shengqi Haotian Biomedical Technology Co., Ltd., Liaoning, Shenyang, China

**Keywords:** T2DM, Flavonoids, oxidative stress, Inflammatory, insulin resistance

## Abstract

The prevalence of type 2 diabetes mellitus (T2DM) is increasing every year. Medications are currently the most common therapy for T2DM. However, these medications have certain adverse effects. In order to find safe and effective ways to improve this disease, researchers have discovered that some natural products can decrease blood sugar. Flavonoids are one of the most essential low molecular weight phenolic chemicals in the plant world, which widely exist in plant roots, stems, leaves, flowers, and fruits. They possess a variety of biological activities, including organ protection, hypoglycemic, lipid-lowering, anti-oxidative and anti-inflammatory effects. Some natural flavonoids ameliorate T2DM and its complications through anti-oxidation, anti-inflammatory action, glucose and lipid metabolism regulation, insulin resistance management, etc. Hence, this review aims at demonstrating the potential benefits of flavonoids in T2DM and its complications. This laid the foundation for the development of novel hypoglycemic medications from flavonoids.

## 1. Introduction

Diabetes Mellitus (DM) is a non-infectious chronic disease caused by malfunction in glucose and lipid metabolism. The progression of disease can result in lesions in organs, major arteries, and microvessels. According to the International Diabetes Federation (IDF), 110 persons aged 20 to 79 has DM, amounting to 537 million people. T2DM is the most common, accounting for 85.0–95.0% of total DM prevalence. Therefore, prevention and treatment of T2DM effectively is a hot topic. The risk factors for T2DM include those associated with lifestyle, such as unhealthy eating, as well as genetic factors that interact with each other and an individual’s living environment (1). Currently, medicine therapy is generally selected in the treatment of T2DM. However, these hypoglycemic medicine have certain toxic and side effects. Thus, experts are exploring for more efficient and risk-free methods of reducing glucose. Flavonoids, the functional components of various natural medicinal plants, have been demonstrated to safely regulate blood glucose levels. They can improve T2DM and its complications through anti-oxidation, anti-inflammatory activity, glucose and lipid metabolism regulation, insulin resistance reduction, etc.

**Figure 1:**
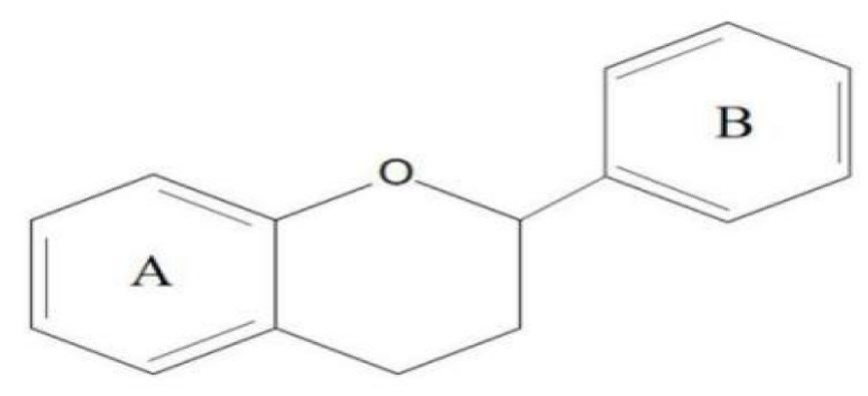
The basic structure of flavonoids.

Flavonoids are one of the most significant low molecular weight phenolic chemicals in the plant world, which mainly in the form of glycosides or carbohydrate groups. They are a series of natural compounds formed of two benzene rings (A and B rings) linked by three carbon atoms, precisely C6-C3-C6 as the generic term for a class of compounds with the basic skeleton (Figure 1), in which C6 is an aromatic C ring and C3 is a heterocycle (2). Flavonoids possess a wide range of biological benefits, including organ protection, hypoglycemic, lipid-lowering, anti-oxidative, and anti-inflammatory properties (Table 1). Therefore, flavonoids have received a lot of interest in the realm of drug research and development.

**Table 1:** Flavonoids.

Type	Flavonoids	Structure	Mechanism	Reference
Flavonols	Quercetin	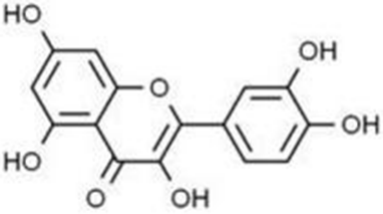	Cardiovascular and cerebrovascular protection, anti-oxidation, immunomodulatory, hypoglycemic, antiviral and anti-inflammatory effects.	(105)
	Rutin	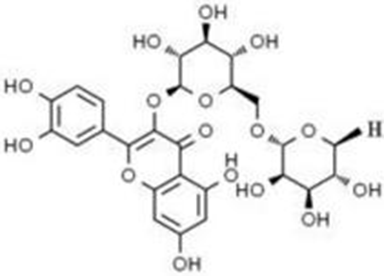	Anti-oxidation, antidiabetic, vasoprotective, neuroprotective and anti-inflammatory effects.	(106–108)
	Kaempferol	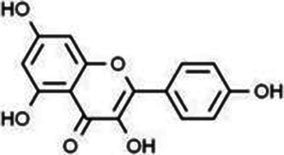	Anti-oxidation, anti-inflammatory, anti-diabetic and anti-aging effects.	(11, 109, 110)
	Isorhamnetin	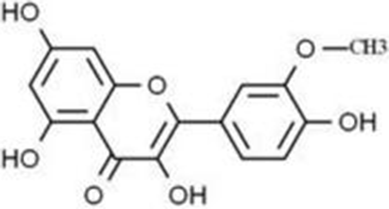	Organ protection, cardiovascular and cerebrovascular protection, prevention of obesity, anti-oxidative and anti-inflammatory effects.	(111)
	Fisetin	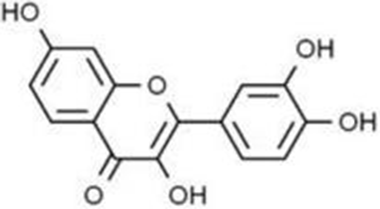	Anti-inflammatory, antioxidant, anti-tumor, and protective effects against myocardial and kidney injury.	(89)
Flavanones	Hesperidin	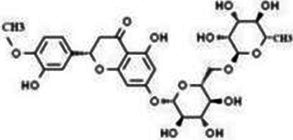	Anti-oxidation, anti-inflammatory, antidiabetic, and cardiovascular protective effects.	(112)
	Naringin	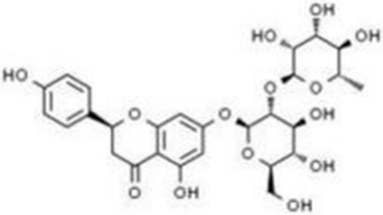	Anti-oxidation, antiadiposity, anti-inflammatory, antidiabetic, antiatherogenic and anti-osteoporotic effects.	(113, 114)
	Naringenin	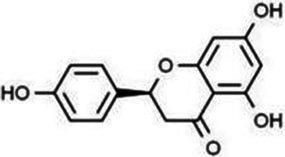	Lipid lowering and insulin-like effects	(15)
Flavanonols	Silymarin	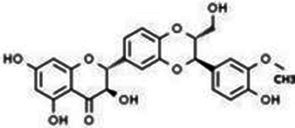	Anti-oxidation, decreasing insulin resistance, cardioprotection, neuroprotection, regulating blood pressure and lipid profile, protecting the pancreas, anti-diabetic and anti-inflammatory effects.	(115–117)
Anthocyanins	Anthocyanin	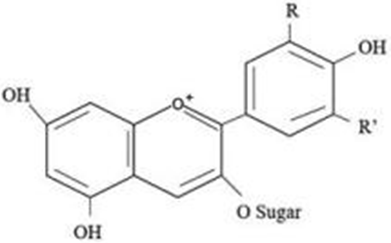	Anti-oxidation,cardiovascular protection, neuroprotection, antiobesity, antidiabetic and anti-inflammatory effects.	(118, 119)
Flavones	Baicalin	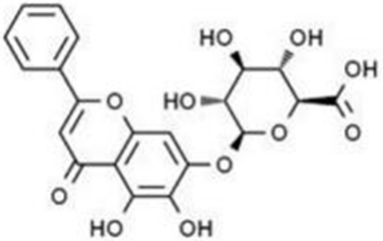	Cardiovascular, hepatic and renal protective, anti-oxidative, anti-infammatory effects.	(120)
	Apigenin	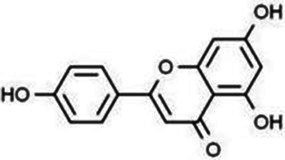	Antimutation, anti-inflammatory, antioxidation, and anticancer effects	(20)
	Luteolin	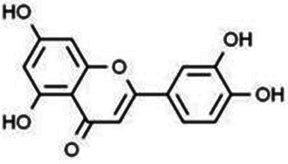	Regulation of glycolipid metabolism, anti-oxidation, neuroprotective, anti-inflammatory and cardioprotective effects.	(35, 121)
	Scutellarin	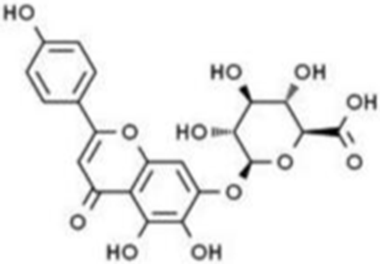	Anti-insulin resistance, anti-inflammatory, anti-obesity and lipid-lowering effects.	(18)
	Myricetin	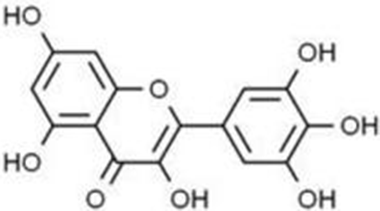	Anti-inflammatory, antitumor, antibacterial, antiviral, and anti-obesity effects	(122)
Isoflavones	Puerarin	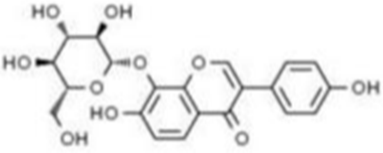	Decreasing blood glucose levels, improving insulin resistance, protecting islets, cardio-protective, anti-oxidative and anti-inflammatory effects.	(123)
	Biochanin A	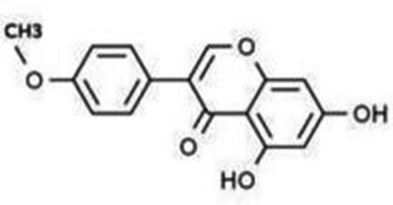	Anti-oxidation, anti-inflammatory and hypoglycaemic effects	(25)
Flavan-3-ol	(−)-Epigallocatechin-3-gallate (EGCG)	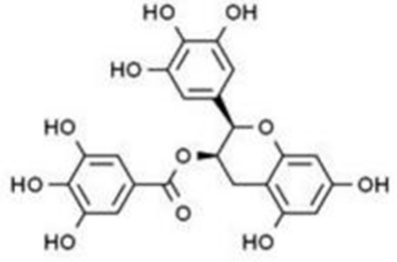	Anti-insulin resistance, anti-obesity and antioxidant effects.	(13)

Recent research has discovered that a number of natural flavonoids isolated from plants can safely and efficiently prevent and cure T2DM and its complications. Chen et al. (3) confirmed that anthocyanins can prevent T2DM by regulating glucose and lipid metabolism. Li et al. (4) found that luteolin can improve diabetic cardiomyopathy by inhibiting inflammation. Sharma et al. (5) proved that kaempferol can decrease diabetic nephropathy by antioxidative effect. Liu et al. (6) detected that puerarin can regulate blood glucose by inhibiting gluconeogenesis. As a result, the purpose of this review is to demonstrate the possible advantages of flavonoids in T2DM and its complications. This laid the foundation for the development of new hypoglycemic drugs from flavonoids.

## 2. The mechanism of flavonoids on T2DM

### 2.1. Flavonoids improve T2DM by decreasing insulin resistance

**Figure 2:**
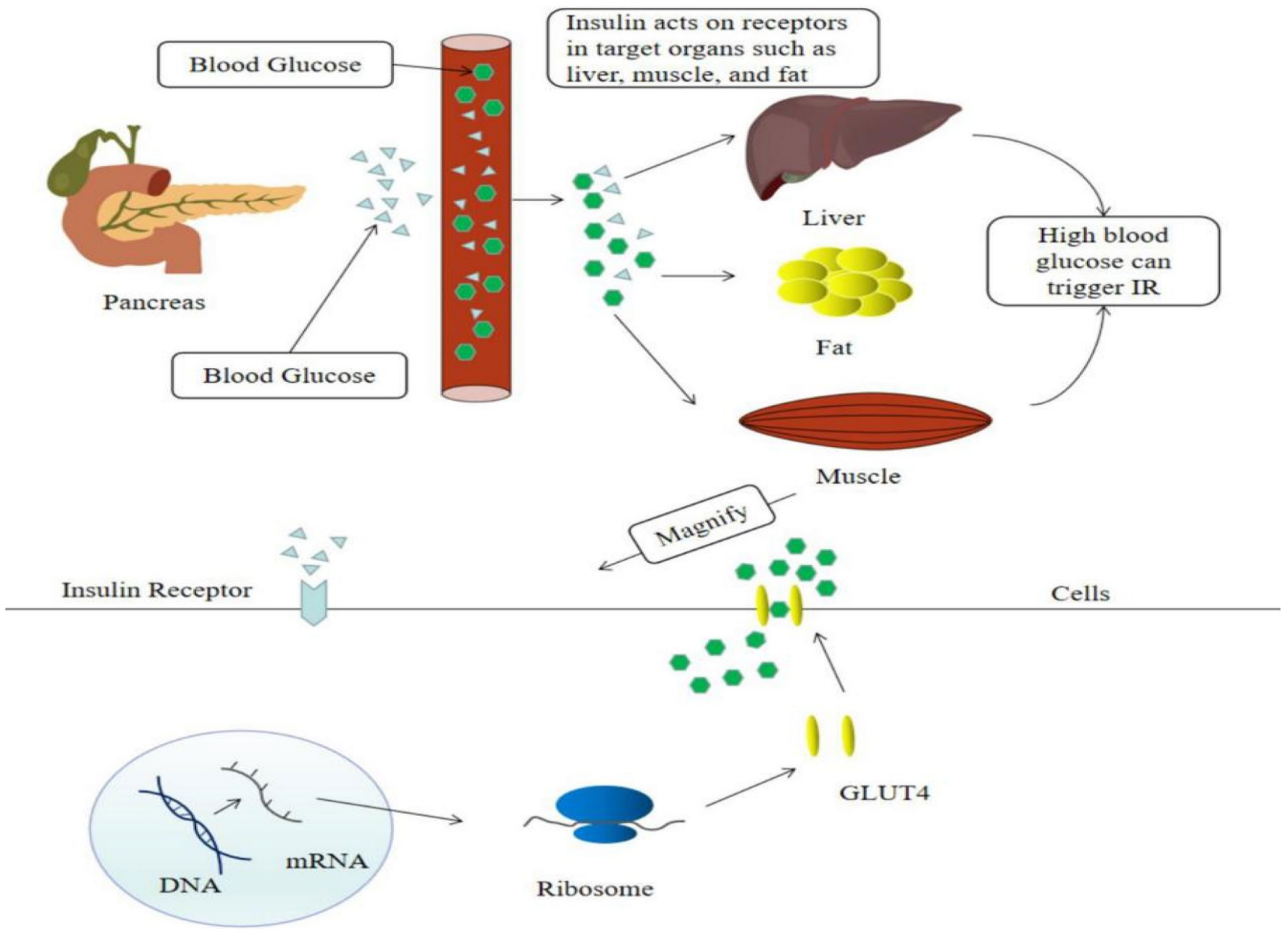
High blood sugar levels cause insulin resistance.

Insulin resistance is a major factor of T2DM. Hyperglycemia causes islet cells to release insulin into the bloodstream, which activates insulin signaling on the surface of cell membranes in target organs such as the liver, muscle, and fat, and induces the translocation of the insulin-dependent Glut4 protein from the cell to the cell surface, facilitating glucose uptake by target cells (7) (Figure 2). Recent evidence suggested that flavonoids cure T2DM by reducing insulin resistance.

The following summary some common flavonoids and explores into the specific mechanisms through which they treat T2DM by reducing insulin resistance deeply. Puerarin, a major active isoflavone extracted from the root of Pueraria lobate, is gaining attention for their potential use in the treatment of T2DM and accumulating evidence has shown that it significantly improves insulin resistance in diabetic animals. Chen et al. (8) discovered the effectiveness of Puerarin on skeletal muscle insulin sensitivity is owing in part to the fact that Puerarin directly stimulates the μ-opioid receptor in skeletal muscle, enhancing insulin signaling. Gao et al. (9) investigated the involvement of fetuin B-AMPK/ACC in the regulation of Puerarin’s influence on insulin resistance in T2DM mice and discovered that Puerarin can reduce insulin resistance in T2DM mice *via* regulating the B-AMPK/ACC signaling pathway. The previous studies showed that baicalin can alleviate insulin resistance, however the particular mechanism of baicalin has not been thoroughly explored. Yu et al. (10) conducted baicalin can prevent insulin resistance and metabolic dysfunction primarily through stimulating the GALR2-GLUT4 signal pathway. It might be a novel method for treating insulin resistance and T2DM in the clinic. Anthocyanins are water-soluble pigments that perform a variety of functions, particularly in the prevention of T2DM (11). Anthocyanins from Lycium ruthenicum, as according Tian et al. (12), can alleviate insulin resistance *via* activating the IRS-1/AKT pathways. A main nutraceutical component of green tea, EGCG, has been recognized as a nutraceutical with effective anti-obesity and antioxidant biological activities. Mi et al. (13) discovered that EGCG prevented TNF-α-induced insulin signaling pathway blockage by reversing redox imbalance and mitochondrial malfunction. Liu et al. (14) observed that EGCG, quercetin or their combination could decrease insulin resistance in HepG2 cells treated with palmitic acid and C57BL/6 mice given a high-fat, high-fructose diet. Naringenin is a flavonoid produced from citrus and grapes that has both lipid-lowering and insulin-like features and could reduce insulin resistance. Jia et al. (15, 16) reported that in HCV core protein (HCVCP) infected mice livers, naringenin had an insulin sensitization effect. In addition, they performed *in vitro* experiments to investigate the insulin sensitization impact of naringenin on HCVCP-infected Huh-7.5.1 cells with IRE1 overexpression or knockdown and determined that naringenin prevented HCVCP-induced endoplasmic reticulum stress, reduced IRE1 downstream gene expression and splicing activity, and prevented IRE1 expression, which resulted in improved insulin resistance. Naringin, a naturally occurring component in citrus fruits, alleviates insulin resistance and improves skeletal muscle atrophy. Termkwancharoen et al. (17) revealed that in high-fat-diet-induced obese rats, naringin is an alternative supplement that improves the treatment of dyslipidaemia, decreases obesity-induced muscular dysfunction, and reduces insulin resistance while increasing sensitivity. Scutellarein, an active component in Erigeronbrevis-capus (Vant.), shows anti-insulin resistance property. According to Gao et al. (18), scutellarein treatment could anti-insulin resistance and anti-oxidative stress. These properties were linked to weight reduction and ameliorated glycometabolism. The underlying mechanisms may be linked to the activation of the insulin signaling pathway and AMP-activated protein kinase (AMPK). Luan et al. (19) suggest that as a modulator of mammalian target of rapamycin (mTOR), scutellarein improves hepatic insulin resistance *via* modulating hepatocyte lipid metabolism through sterol regulatory element-binding protein 1c (SREBP-1c) inhibition. Apigenin, a nontoxic and naturally occurring flavonoid, has been shown to alleviate insulin resistance. Wu et al. (20) revealed that apigenin might diminish insulin resistance *via* modulating endoplasmic reticulum stress by decreasing endoplasmic reticulum stress related proteins. Luteolin is a flavonoid contained in many vegetables, herbs and fruits, which is a medicine for improving insulin resistance. Huang et al. (21) demonstrated that luteolin reduced insulin resistance *via* activating the PI3K/AKT signaling pathway. Baek et al. (22) indicate that dietary luteolin supplementation may reduce adipose tissue insulin resistance by reducing M1-like polarization of macrophages in adipose tissue.

Several uncommon flavonoids have also been discovered to regulate T2DM by improving insulin resistance. Isorhamnetin, a potential pharmaceutical option for the treatment of T2DM, ameliorates insulin resistance, inflammation, and oxidative stress (23). Matboli et al. (24) intended to investigate the mechanism for the effect of isorhamnetin that regulates the route of insulin signaling, and demonstrated that through altering the insulin resistance signaling pathway-related RNA network, isorhamnetin might be utilized as an prospective supplemental therapy in T2DM. The expression of m-TOR mRNA, IGF1-R, and LncRNA-RP11-773H22.4 is downregulated by isorhamnetin, whereas the expression of AKT2 mRNA, miR-1, and miR-3,163 is upregulated. In rats with diabetes caused by a high-fat diet, biochanin A, a soy isoflavone, reduces insulin resistance through altering the insulin signaling pathway. According to Arjunan et al. (25), biochanin A therapy dramatically enhanced the phosphorylation levels of the skeletal muscle proteins GLUT4, phosphatidylinositol-3-kinase (PI3K), IRS-1, and p-AKT. This demonstrates unequivocally that biochanin A is effective in successfully triggering a glucose transport by GLUT4 migration and restoring glucose sensitivity. By regulating PI3K/Akt signaling, biochanin A also could enhance insulin sensitivity.

In summary, this part discusses numerous flavonoids in the treatment of T2DM by reducing insulin resistance, as well as their improvement mechanisms, providing an accurate theoretical basis for the clinical development of new hypoglycemic medicines. However, because there are so many different flavonoids that can improve insulin resistance, more research is necessary to further analyze the differences between them and explore their potential value.

### 2.2. Flavonoids ameliorate T2DM by reducing oxidative stress

**Figure 3:**
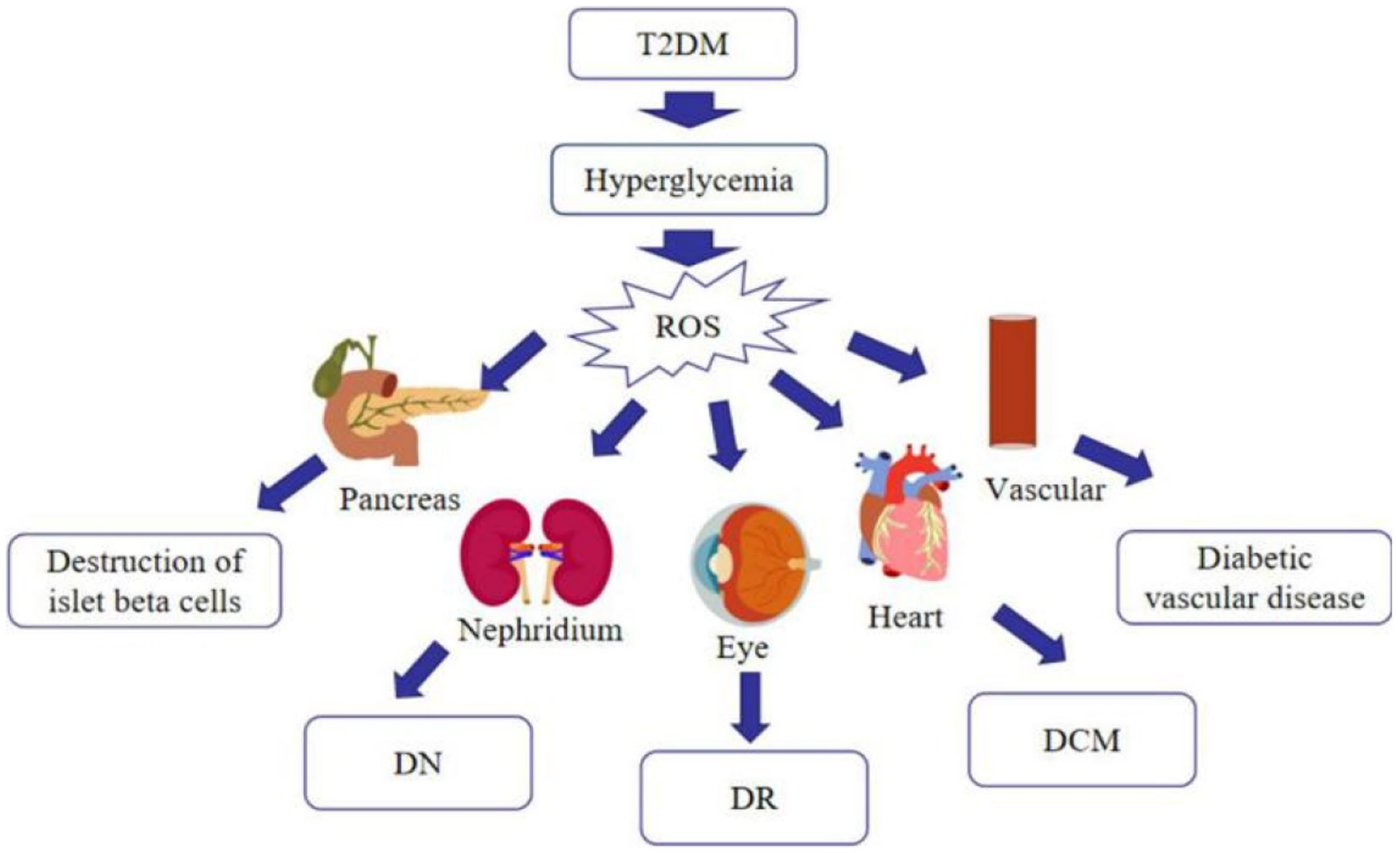
Effects of oxidative stress on T2DM and its complications.

Much emphasis has been spent on researching oxidative stress, since it is now commonly accepted that it is associated with the advancement of T2DM and its complications (26). Hyperglycemia increases mitochondrial dysfunction and the generation of reactive oxygen species (ROS), which causes oxidative stress in various organs, including blood vessels and pancreatic beta cells, as diabetes progresses. Stress-responsive intracellular signaling molecules are triggered in the absence of compensating mechanisms, resulting in cellular damage. Elevated intracellular ROS levels and consequent oxidative stress play a significant role in the development of vascular complications of diabetes. Furthermore, advanced glycation end products (AGEs) are formed as a result of the non-enzymatic covalent attachment of glucose and its toxic derivatives to biological macromolecules. The presence of AGEs in the tissue enhances the production of pro-inflammatory cytokines and the creation of free radicals. Because of the persistent exposure of cells to high glucose levels, glucose toxicity may occur in multiple organs, leading to a variety of diabetic complications (27) (Figure 3). As just a result, anti-oxidation may be an important strategy in the treatment of T2DM. Currently, studies have revealed that many flavonoids in nature contain anti-oxidative effect.

*Citrus reticulata* fruit peel has been shown to have significant levels of hesperidin and quercetin. Ali et al. (26) conducted the anti-diabetic effects of hesperidin and quercetin and discovered that hesperidin and quercetin from citrus reticulata fruit peel can improve the antioxidant defense system manifested in nicotinamide/streptozotocin (STZ)-induced T2DM rats *via* increasing the glutathione content and activity of GPx, GST, and SOD and decreasing the liver lipid peroxidation. Anti-oxidant activities of puerarin have been demonstrated. Chen et al. (28) researched the influence of puerarin on the function of mitochondria in diabetic rats and discovered that puerarin can protect mitochondria in muscle from oxidative damage by reducing the phosphorylation of p66Shc and enhancing the expression levels of SIRT3, SOD2, UCP2, and UCP3, thereby increasing the effectiveness of antioxidant defense and decreasing ROS production. In the bloodstream, it can also prevent oxidative stress by increasing total SOD activity and decreasing MDA levels. Anthocyanin has a potent antioxidant activity, which serves to lower the risk of diabetes. Herrera-Balandrano et al. (29) observed that high glucose dramatically elevated hepatic oxidative stress, which raised ROS by up to 6-fold and impaired cell viability. However, blueberry anthocyanin extract (BAE) possesses a strong antioxidant capacity that can significantly protect hepatic cells from oxidative degradation by enhancing SOD antioxidant activity, decreasing ROS production, and improving cell viability. According to Liu et al. (30), Anthocyanins from *Padus racemosa* (APR) could effectively reduce production of ROS, promote cell viability, and block the activation of the redox-sensitive pathways factor-kappa B (NF-κB) and mitogen-activated protein kinase (MAPK), which might be triggered by ROS. Naringin and hesperidin, extracted from the ordinary orange *Citrus aurantium* and other species of the genus Citrus, may ameliorate hyperglycemia-induced oxidative stress. Mahmoud et al. (31) discovered that hesperidin and naringin, through their antioxidant capabilities, improved serum and liver NO, MDA and glutathione, as well as liver antioxidant enzymes, and increased antioxidant defense system activity, conferring protection against HFD/STZ-induced T2DM in rats. Antioxidant effects of quercetin treatment have been shown in STZ-induced diabetic rats. According to Mahesh et al. (32), who investigated in STZ-induced diabetic rats, the effect of quercetin therapy on nonenzymic antioxidant defense and activities of circulatory enzymic, quercetin significantly reduced lipid hydroperoxide, vitamin E, and plasma TBARS levels while significantly increasing plasma vitamin C levels. Changes in CAT and SOD levels were also observed. These findings suggest that quercetin reduced the oxidative stress caused by diabetes. Myricetin can be extracted in *Syzygium cumini* (L.). Chagas et al. (33) investigated the anti-diabetic effects of *S. cumini* leaf extracts, and discovered that specific antioxidant activity of they are assessed against both DPPH^•^ and ABTS^•+^, as well as they also prevented lipoxygenase activity, and then confirmed that these extracts can imrove oxidative Stress-induced diabetic rats. Naringenin, a natural component that is both anti-inflammatory and antioxidant, reduces inflammation-mediated nitric oxide overproduction as well as enhances endogenous antioxidant status during hyperglycemia. Rehman et al. (34) revealed that hyperglycemia might be the reason for the increased oxidative stress caused by a decrease in SOD and an increase in NO levels. Naringenin inhibits inflammatory mediators such as TNF-α and IL-6, hence controlling NO generation and increasing SOD antioxidant levels. All of this contributes to ameliorate the compromised status of glycemia by reducing inflammation and NO-mediated oxidative stress.

### 2.3. Flavonoids improve T2DM by regulating glycolipid metabolism disorders

Glycolipid metabolism disorder (GLMD) is a form of metabolic syndrome defined by abdominal obesity and abnormal lipid and carbohydrate metabolism in the blood, including T2DM, obesity, insulin resistance, hyperlipidemia, etc. Many treatments, which including bariatric surgery, and weight loss drugs, are now employed to treat diabetic lipid/metabolic disorders. However, these tactics have undesirable side effects such as gastrointestinal symptoms, memory difficulty, and fatigue. As a result, finding healthful and safe phytochemicals that may ease the GLMD effects of diabetes and obesity is critical (35). Several studies have established that certain flavonoids found in nature have the capability to regulate GLMD.

Black soybean seed coat extract (BSSCE) is abundant in anthocyanins, which have a variety of health benefits. Chen et al. (3) reported that BSSCE could dramatically alleviate hyperglycemia and hyperlipidemia in diabetic mice by decreasing insulin resistance, blood glucose levels, serum lipid levels, and increasing antioxidant activities of SOD, GPX, and CAT in liver tissue, the glucose tolerance. Rutin, as a flavonol glycoside, has been shown to significantly improve GLMD. Sun et al. (36) identified that rutin has considerable hypoglycemic effect and enhances insulin sensitivity. Rutin also boosted CAT, SOD, and GSH-Px levels while reduced MDA levels, emphasizing its anti-oxidant action. It also caused a considerable decrease in TG, TC and LDL-C levels, in addition to an increase in HDL-C levels. Silymarin is an anti-diabetic flavonoid oligosaccharide molecule. Silymarin has been found to modulate glucose and lipid metabolism by modulating two critical proteins, SREBP-1c and Sirtuin1 (SIRT1). SIRT1 acts as a master switch, controlling critical metabolic regulators such as SREBP-1c to maintain lipid and glucose homeostasis, insulin secretion and sensitivity, and energy balance. SREBP-1c is a transcription factor that controls genes in the glycolysis and *de novo* lipogenesis pathways. In rats with T2DM, Feng et al. and Kheiripour et al. (37, 38) clearly showed that silymarin could reduce oxidative stress, upregulate SIRT1, and decrease SREBP-1c gene expression. In addition, silymarin elevated glycogen content, prevented lipid accumulation, and improved hepatic insulin resistance.

In summary, certain flavonoids have the ability to regulate glucose and lipid metabolism, which can effectively reduce the onset and progression of diabetes, particularly obese T2DM. Nevertheless, dietary management is a crucial aspect of managing obesity-related T2DM. Flavonoids are known to interact with other nutrients in the diet, such as vitamins and minerals, which can affect their absorption and bioavailability. Therefore, it is essential to consider the potential interactions between flavonoids and other nutrients when evaluating their efficacy in treating T2DM.

### 2.4. Flavonoids regulate T2DM through controlling gluconeogenesis.

The production of free glucose from non-carbohydrate carbon substrates such as pyruvate, glycerol, lactic acid, and glycogenic amino acids is known as gluconeogenesis (39). Firstly, pyruvic acid is carboxylated by pyruvate carboxylase to generate oxaloacetate. Following that, oxaloacetate is decarboxylated *via* phosphoenol pyruvate (PEP) carboxykinase to create PEP. Subsequent stages are glycolytic processes in reverse and result in forming dihydroxyacetone phosphate and glyceraldehyde 3-phosphate. Aldolase combines these two molecules to generate fructose 1,6-bisphosphate. The enzyme fructose 1,6-bisphosphatase dephosphorylates fructose 1,6-bisphosphate to generate fructose 6-phosphate, that is isomerized to form glucose-6-phosphate. Glucose 6-phosphatase dephosphorylates glucose 6-phosphate, yielding in glucose (40). Gluconeogenesis has been found in studies to be particularly active in T2DM patients (41), which is also an important reason for increased endogenous glucose production. As a result, controlling gluconeogenesis is critical for managing blood glucose. Currently, research indicates that certain natural flavonoids can reduce blood sugar *via* controlling liver gluconeogenesis.

Kaempferol, a flavonol found in many medicinal herbs, has been shown to ameliorate hyperglycemia by increasing reducing gluconeogenesis in the liver and glucose metabolism in skeletal muscle. According to Alkhalidy et al. (42), oral treatment of kaempferol to diabetic mice reduced the incidence of overt diabetes from 100 to 77.8%. Kaempferol treatment restored hexokinase activity in the muscle and liver of the diabetic mice while inhibiting while suppressing hepatic pyruvate carboxylase activity and gluconeogenesis. Puerarin, the major component of pueraria, a Chinese herbal medication, plays a significant part in several traditional diabetic treatments. The current study is primarily concerned with investigating the probable mechanism of puerarin in suppressing hepatic gluconeogenesis. Puerarin, found by Liu et al. (6), can increase the phosphorylation of forkhead box protein O1 (FOXO1) *via* activating the PI3K/Akt signaling pathway, and it also suppresses the expression of PEPCK and G6Pase, inhibiting gluconeogenesis.

In summary, kaempferol and puerarin can regulate T2DM through modulating gluconeogenesis. However, they must be further studied in order to lay the foundation for the development of new clinical medication. Moreover, there is limited research on the effects of flavonoids on gluconeogenesis, a key factor of glucose metabolism. Therefore, the potential mechanism of flavonoids to improve gluconeogenesis of T2DM remains to be investigated.

### 2.5. Flavonoids ameliorate T2DM by inhibiting α-glucosidase.

Following a meal, the initial stage of carbohydrate breakdown occurs in the mouth, where salivary α-amylase hydrolyzes large polysaccharides to disaccharides. During digestion, the α-amylase products are further broken down into glucose by α-glucosidase in the lumen of the small intestine. Barik et al. (43) observed that anthocyanins, in black currants, can largely reduce postprandial hyperglycemia by suppressing α-glucosidase.

## 3. Complications of T2DM regulated by flavonoids

### 3.1. Flavonoids regulate diabetic vascular disease

**Figure 4:**
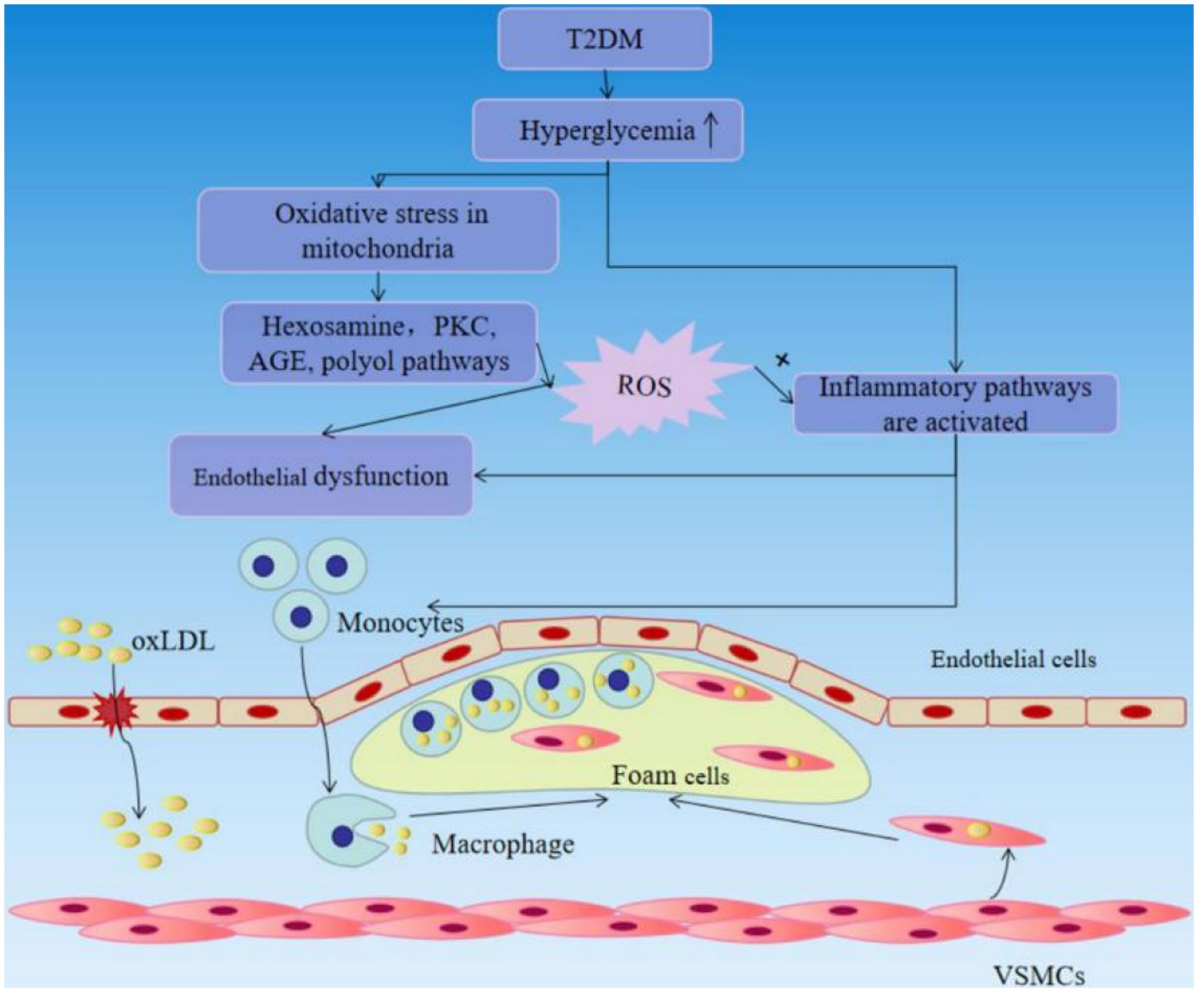
T2DM causes vascular endothelial damage and accelerates the progression of atherosclerosis.

Vascular complications are the leading cause of mortality and disability in diabetic individuals. Endothelial dysfunction is described as the beginning of hyperglycemia-related vascular disorders such as atherosclerosis at an early age. Hyperglycemia can generate a non-classic inflammatory response in the vascular endothelium and contributes to the inflammatory response *via* intracellular ROS aggregation. Hyperglycemia may cause oxidative stress in mitochondria, activating four recognized pathways, including the hexosamine, polyol and protein kinase C (PKC), AGE pathways. These pathways increase the production of ROS, and ROS increase insulin resistance, endothelial dysfunction, the expression of inflammatory and adhesion factors, which promote monocyte and T-cell adhesion to the vascular endothelium and infiltration to form neointima lesions, and the production of oxidized-low density lipoprotein (oxLDL). Moreover, increased AGE may enhance monocyte/macrophage adherence and entry into the subendothelial space. Enhanced PKC signaling has multiple pro-atherogenic consequences, including decreased generation of NO and impaired vasodilation, endothelial dysfunction and increased permeability. The blood vessel endothelium responds to the mechanical stress with activation that subsequently leads to the recruitment of circulating immune cells. Circulating monocytes cling to the wounded portion of the artery wall and penetrate within, where they differentiate into macrophages, which actively engage in lipid absorption *via* phagocytosis and give birth to foam cells (44–46) (Figure 4). Also, a considerable number of foam cells will gather to produce intraclumen projecting arterial plaque, resulting in narrowing of blood vessel inner diameter and inadequate blood flow. As excessive foam cells form, the fiber cap thins and ruptures, leading the recruitment of circulating platelets to the wounded site and stimulating the synthesis of thrombin and fibrin, resulting in thrombosis and the development of various arterial illnesses such as myocardial infarction and stroke (47). As can be seen, the harm caused by late diabetes vascular disease to the human body cannot be overlooked, therefore we must pay close attention to diabetic vascular disease therapy.

For many years, puerarin has been used to treat cardiovascular diseases in China extensively. Puerarin has been proven in studies to have enormous potential and particular therapeutic usefulness in hyperglycemia-related cardiovascular disease and the creation of novel medications. Puerarin protects against hyperglycemia-associated inter-endothelial junction disruption, according to Lian et al. (44) It may restore inter-endothelial junction disruption by blocking Nod-like receptor protein 3 (NLRP3) inflammasome activation, decreasing subsequent caspase-1 activation, and activating the release of high mobility group box 1 (HMGB1) by lowering intracellular ROS levels. Silymarin possesses antioxidant and therapeutic effects, as well as the ability to impact angiogenesis. Stolf et al. (48) showed that silymarin therapy partially alleviated the inflammatory response and deficits in oxidative defenses and angiogenesis induced by STZ-induced diabetes in mice. Silymarin is likewise thought to be reasonably safe, having a low rate of adverse effects. Silymarin may be a candidate for treating diabetic complications, considering the adverse effects of medicine widely employed as adjuvants in diabetes treatment. By lowering oxidative stress, rutin can protect vascular smooth muscle cells from early senescence and stabilizes atherosclerosis in diabetic rats. According to Li et al. (49), rutin dramatically reduces glucose and lipid metabolic disturbances in diabetes. Besides that, rutin reduced the atherosclerotic burden and senescent cell number while increasing the vascular smooth muscle cell (VSMCs) ratio in aortic root plaque. *In vitro*, rutin reduced premature senescence caused by oxidative stress, and that the protective action may be achieved by reducing oxidative stress and preserving telomeres. Rutin treatment reduces the burden of atherosclerosis and stabilizes plaque *via* reducing metabolic disruption and preventing VSMC senescence. Diabetes-induced endothelial dysfunction was alleviated by quercetin supplementation through inhibiting the endoplasmic reticulum stress pathway. In STZ-induced diabetic rats, Suganya et al. (50) observed that quercetin increased insulin production and lowered blood glucose levels. According to immunohistochemistry examination, quercetin decreased pancreatic ER stress-induced endothelial dysfunction. Also, in diabetic rats, quercetin treatment raised the expression of vascular endothelial growth factor (VEGF) and its receptor, VEGFR2.

In summary, flavonoids can prevent diabetic vascular disease by anti-oxidation, surpressing inflammatory response, and inhibiting the endoplasmic reticulum stress pathway. Even so, the optimal dosage, duration, and formulation of some flavonoids for achieving these effects remain unclear. Therefore, further research is needed to elucidate the mechanisms underlying the therapeutic effects of flavonoids on diabetic vascular complications and to optimize their use in clinical practice.

### 3.2. Flavonoids improve diabetes cardiomyopathy

Diabetic cardiomyopathy (DCM) was defined as a disease characterized by heart failure in the lack of valvular heart disease, coronary artery disease, or hypertension (51). The increased formation of AGEs secondary to hyperglycaemia can form cross-linking within or between proteins by covalently combining with other AGEs. Extracellular proteins that are stable are vulnerable to the accumulation of AGE crosslinks. This may impair collagen’s ability to degrade, resulting in collagen accumulation and fibrosis, increasing myocardial hardness and impairing diastolic function (52). Moreover, AGEs activate transforming growth factor β (TGF-β) *via* the AGE/RAGE pathway, and superoxide produced by the combination of TGF-β and NADPH oxidase can mix with NO to form ROS, which can harm cardiac cells (53). Diabetes is a state of pro-inflammatory. Increased production of inflammatory cytokines (TGF-β1 and TNF-α), expression of cell adhesion molecules (VCAM-1 and ICAM-1), and infiltration of macrophages and leucocytes in DCM. The significance of oxidative stress in the progression of DCM is extensively acknowledged. ROS can induce oxidative myocardial injury by directly damaging proteins or phospholipids in myocardial cells (54). Diabetes patients have aberrant insulin metabolic signaling, which reduces insulin-stimulated cardiac nitric oxide (NO) generation and endothelial nitric oxide synthase (eNOS) activity while increasing cardiomyocyte intracellular Ca^2+^/Ca^2+^ sensitivity and decreasing sarcoplasmic Ca^2+^ absorption. These flaws lead to the development of intracellular calcium overload, which results in increased myocardial stiffness and poorer relaxation, both of which are hallmarks of DCM (55). Flavonoids can ameliorate DCM mainly through acting as anti-inflammatory and anti-oxidation effects.

Silymarin, kaempferol, quercetin, puerarin, Luteolin, and rutin are among the flavonoids that have received the most attention for their potential to improve DCM. Silymarin might be a possible target for DCM therapy. Meng et al. (56) demonstrated that silymarin reduced blood glucose levels while greatly improving cardiac fibrosis and collagen deposition in diabetic rats. According to the echocardiography data, silymarin treatment reduced heart dysfunction in diabetic rats. Moreover, as compared to untreated diabetic rats, silymarin treatment reduced TGF-β1 and p-Smad2/3 levels while increasing Smad7. Silymarin improves DCM *via* inhibiting TGF-β1/Smad signaling. In diabetic rats, kaempferol might reduce the oxidative, inflammatory, and fibrotic damage to the left ventricles (LVs). Alshehri et al. (57) established that kaempferol had a cardioprotective effect in diabetic rats. The administration of kaempferol to diabetic rats greatly protected the systolic and diastolic functioning of the LVs, and the mechanism of protection is owing to powerful anti-inflammatory and antioxidant actions mediated through activation/up-regulation of SIRT1. The usage of quercetin may assist in lowering cardiovascular risk in T2DM patients. Gorbenko et al. (58) showed that quercetin reduced free radical oxidation in the myocardial mitochondria of T2DM rats, decreasing the synthesis of advanced oxidation protein products (AOPP). Furthermore, quercetin at 50 mg/kg and 10 mg/kg doses reduced the development of oxidative stress in the heart of diabetic rats. Puerarin had cardioprotective benefits on DCM by decreasing inflammation, and it may be a promising approach for DCM therapy. Yin et al. (59) revealed that puerarin may have promising therapeutic potential for diabetic cardiovascular problems, with the mechanism perhaps being the reduction of diabetes-induced cell death, fibrotic, and inflammation pathways. Puerarin also substantially reduces the generation of pro-inflammatory cytokines in diabetic rats and H9C2 cells *via* inhibiting NF-κB pathways. Luteolin has cardioprotective properties and might be an effective treatment for DCM. Li et al. (4) discovered that luteolin Ameliorates DCM through inhibiting NF-κB-mediated inflammatory and nuclear factor erythroid-derived-2-like 2 (Nrf2)-mediated oxidative stress responses, decreasing hypertrophy, fibrosis, and apoptosis. The use of therapeutic rutin may enhance myocardial function. Huang et al. (60) found that rutin overturned myocardial hypertrophy, alleviated lipid accumulation and collagen deposition, and increased capillary density in diabetic myocardial tissues, and massively improved cardiac function as well as glucose and lipid metabolism by activating the Akt and MAPK pathways and lowering oxidative stress.

**Figure 5:**
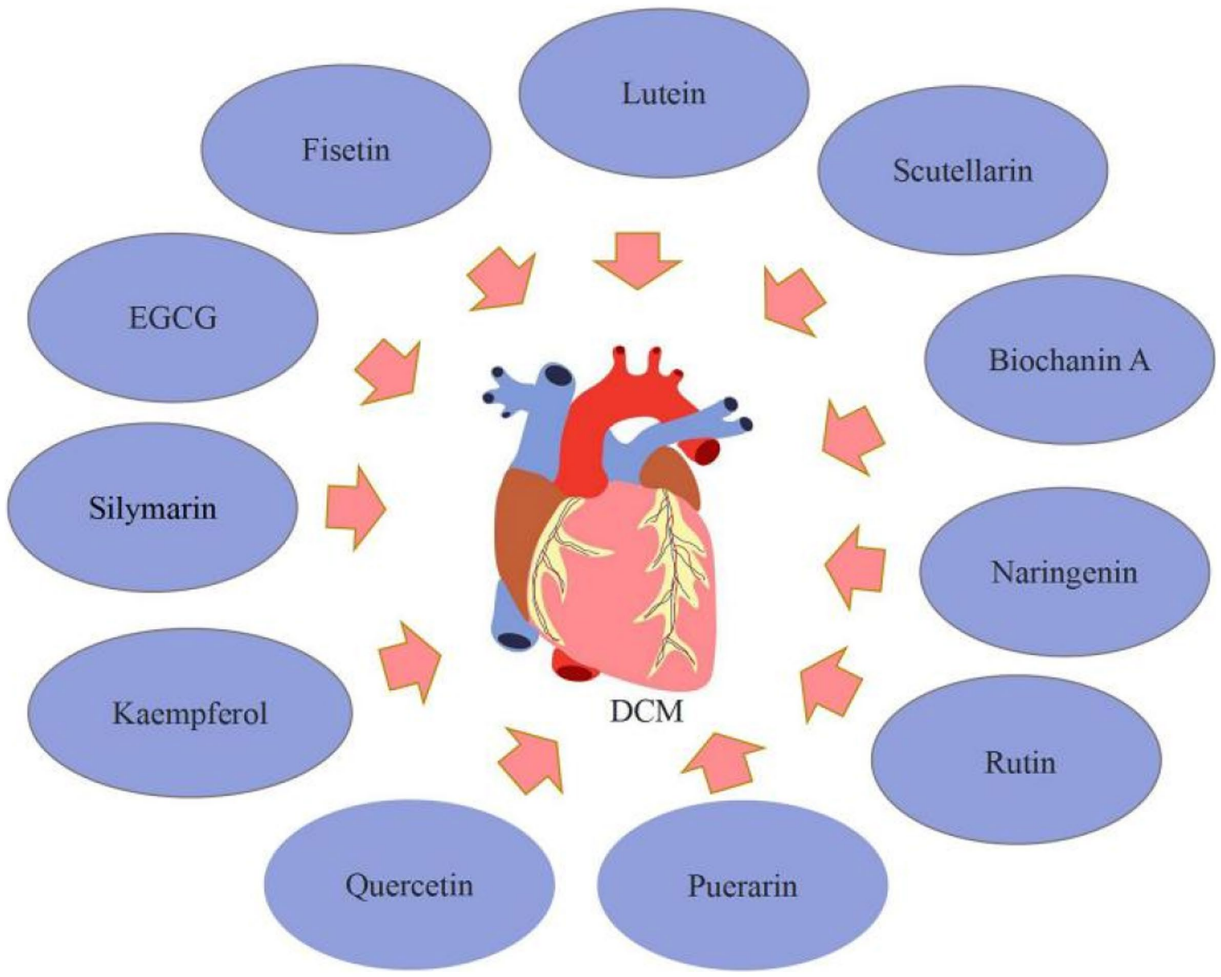
Flavonoids for the treatment of DCM.

Additionally, EGCG, naringenin, scutellarin, and fisetin were discovered to enhance DCM in the summary. EGCG has anti-fibrotic properties in a variety of tissues and has been shown to reduce cardiac fibrosis in T2DM rats. Diabetes, according to Jia et al. (61), severely reduced cardiac contractile function and exacerbated myocardial hypertrophy and injury. Diabetes also inhibited autophagy activation in myocardial tissue and caused cardiac fibrosis. In the EGCG treatment groups, different dosages of EGCG reduced myocardial contractile dysfunction, hypertrophy, and injury; myocardial autophagy was activated; and myocardial fibrosis was reduced. Furthermore, its underlying mechanisms are connected with autophagy activation through regulation of the AMPK/mTOR pathway, followed by inhibition of the TGF-β/MMPs pathway. Naringenin has cardioprotective effects. He et al. (62) indicated that naringenin decreased cardiomyocyte apoptosis and cardiac fibrosis, as well as blood glucose levels. Naringenin suppressed pro-inflammatory cytokines, the number of ROS, and the expression of NF-κB, yet dramatically increased the expression of Nrf2 and antioxidant enzymes. Also, naringenin substantially decreased cell apoptosis in high glucose-treated H9C2 cardiac cells *via* decreasing the generation of pro-inflammatory cytokines and ROS. Zhang et al. (63) discovered that the mRNA and protein expressions of PPARs were decreased, the level of 14, 15-EET and the protein expression of CYP2J3 were down-regulated following diabetic cardiac hypertrophy. In diabetic mice, Naringenin can ameliorate cardiac hypertrophy by increasing CYP2J3 expression, EET levels, and PPAR expression. Scutellarin, a flavonoid extracted from Erigeron breviscapus (vant.), has a variety of pharmacological properties, such as reducing cardiac fibrosis and the size and dysfunction of infarcts in rats with myocardial ischemia, preventing cardiac hypertrophy, and guarding against acute cardiotoxicity caused by doxorubicin. Scutellarin was shown by Xu et al. (64) to have anti-inflammatory, antioxidant, and antifibrotic actions on diabetic mice and to lessen myocardial damage. AKT and Nrf2/HO-1 activation and NF-κB/NLRP3 signaling pathway inhibition may be connected to the molecular molecular. Scutellarin therapy of T2DM mice at doses of 10 or 20 mg/kg body weight significantly reduced indices of metabolic, lipidemic, and cardiac functioning, according to Huo et al. (65) Additionally, scutellarin reduced inflammation, apoptosis, and oxidative stress in the T2DM heart through activating the Nrf2/Keap1/ARE pathway and suppressing the mitochondrial apoptotic pathway and the TLR/MYD88/NF-κB pathway. Su et al. (66) found that scutellarin may reduce the symptoms of DCM by increasing the autophagy-associated proteins of cardiomyocytes, such as LC3-I, LC3-II, and Beclin-1 and decreasing those linked to apoptosis, such as Cyt-C, Bax, and caspase-3. Biochanin A, a methylated isoflavone present in flowering tops of *Trifolium pratense* can improve T2DM cardiomyopathy. According to research by Oza et al. (67), biochanin A therapy at various dosages may improve cardiomyopathy in T2DM mice through enhancing SIRT1 expression and decreasing oxidative stress. Fisetin, a bioactive flavonol molecule existing in many plants, may be useful in treating human DCM. Althunibat et al. (68) observed that fisetin decreased the development of DCM by reducing hyperglycemia/hyperlipidemia-induced inflammation, oxidative stress, and apoptosis (Figure 5).

In summary, flavonoids, such as silymarin, fisetin, and biochanin A can contribute to DCM. Still, these findings have significant limitations because the experimental investigation concentrated on the anti-inflammatory, antioxidant, antifibrotic, apoptosis, and autophagy pathways of cardiomyocytes, although other pathways are also implicated in DCM. As a result, the effect of flavonoids on additional DCM-related pathways has to be investigated.

### 3.3. Flavonoids ameliorate diabetic nephropathy

Diabetic nephropathy (DN) is the main cause of end-stage renal disease. Continuous proteinuria and gradual deterioration in renal function are the predominant clinical symptoms of DN (69). Hyperglycemia increases the production of ROS. In the glomerular microcirculation, increased ROS generation lowers nitric oxide bioavailability, leading to dysregulation of arteriolar tone and mesangial contraction, as well as persistent oxidative stress, which causes leukocyte adhesion, endothelial dysfunction, and glomerular cell apoptosis (70). Hyperglycemia causes damaged glomerular and tubular cells to produce inflammatory mediators (cytokines and chemokines), which contribute to renal injury through a variety of mechanisms, including podocyte/tubular damage, mesangial proliferation, and leukocyte infiltration (71). Glomerular mesangial cells (GMCs) keep the renal mesangial matrix and glomerular capillaries structurally stable. By increasing CHOP production, high hyperglycemia causes endoplasmic reticulum stress in GMCs, which leads to cell necrosis (72). In addition, DN is also related to miRNAs, exosomes and epigenetics pathways (73). Flavonoids, which operate as anti-oxidation, anti-inflammatory, and anti-fibrogenic effects, can heal renal damage and improve renal function, making them viable medications for the prevention and treatment of DN.

Numerous investigations have established that luteolin has anti-inflammatory and antioxidant properties, which may have a significance in kidney preservation in DN patients. Zhang et al. (74) observed that in DN mouse models, by decreasing the oxidative stress and inflammatory response, Luteolin might alleviate glomerular sclerosis and interstitial fibrosis. And it may perform its biological function primarily by suppressing STAT3 activity. What’s more, Xiong et al. (75) demonstrated that luteolin could preserve basement membrane filtration function *via* upregulating Nphs2 protein expression and preventing apoptosis, fusion, and deletion of podocytes under high glucose circumstances. Besides which, luteolin may suppress glomerulosclerosis while maintaining the relatively normal physiological structure of glomeruli, implying that luteolin might slow the progression of DN. Rutin may inhibit the advancement of diabetic nephropathy by improvement of metabolic acidosis and fbrosis. In alloxan-induced diabetic rats, Ganesan et al. (76) reported that rutin inhibited urinary ketone body production and lowered the level of urea, serum creatinine, cholesterol and serum triglycerides,and prevent diabetic ketoacidodis and fbrosis. Baicalin is a possible natural substance that tackles oxidative stress and inflammation to treat DN. Ma et al. (77) found that baicalin can alleviate DN by reducing oxidative stress and inflammation, and its essential mechanisms were linked to the activation of the Nrf2-mediated antioxidant signaling pathway and the suppression of the MAPK-mediated inflammatory signaling pathway. Given the close relationship between baicalin, miR-124, TLR4, inflammation, and renal fibrosis, Zhang et al. (78) looked into the link between baicalin and the miR-124/TLR4/NF-κB axis and discovered that by inhibiting TLR4/NF-κB pathway and upregulating miR-124, baicalin alleviated renal fibrosis. Nam et al. (79) determined that in diabetic human renal proximal tubular cells, baicalin inhibited the transactivation of STAT3 and NF-κB as well as the related inflammatory and fibrosis markers. Moreover, GABAAR, as a potential receptor for baicalin, mediates anti-fibrogenic actions of baicalin by decreasing intracellular Ca^2+^ concentration and, as a result, inhibiting NF-κB and STAT3 activation. Anthocyanins have been shown to have significant antioxidative activities in animal models of diabetes mellitus and to protect against kidney injury induced by hyperglycemia. Wei et al. (80) found that in diabetic mice kidneys, anthocyanins inhibited the expression of thioredoxin interacting protein (TXNIP) while increasing extracellular signal-regulated kinase 1/2 (ERK1/2) and p38MAPK activity and suppressing renal cellular death. Puerarin has renoprotective effects in diabetics, significantly reducing albuminuria and diabetic kidney damage. In a mouse model with established DN, Li et al. (81) confirmed the renoprotective actions of puerarin administration, and showed that puerarin, a stimulator of SIRT1 expression in podocytes, exerts anti-oxidative properties *via* activating SIRT1-mediated NF-κB deacetylation. Kaempferol suppresses RhoA/Rho Kinase and has the potential to be employed as a medicinal agent to treat kidney damage caused by diabetes. According to Sharma et al. (5), kaempferol suppresses hyperglycemia-induced RhoA activation and reduces pro-inflammatory cytokines, oxidative stress, and fibrosis in NRK-52E and RPTEC cells. Hesperetin has the ability to minimize kidney damage, restore kidney function, and postpone DN. Zhang et al. (82) clearly revealed that hesperetin restored the function of podocytes, inhibited the TGF-β1-ILK-Akt signaling pathway, and reduced abnormal alterations of podocyte surface protein expression. Chen et al. (83) found that hesperetin significantly improved the renal functions and structural alterations of diabetic rats, as well as up-regulation of glyoxalase 1 (Glo-1) and suppression of inflammation and the AGEs/RAGE axis. Moreover, hesperetin massively increased Nrf2 and p-Nrf2 levels, and also caused up-regulation of γ-glutamylcysteine synthetase, which is the target gene of Nrf2/ARE signaling. In early DN, quercetin can increase the proliferation of glomerular mesangial cells (MCs). Lei et al. (84) observed that quercetin treatment improved renal function, renal fibrosis, and lipid levels. The Hippo pathway was inactivated in high glucose high-glucose- (HG)-induced MC proliferation, and quercetin inhibited proliferation by reactivating the Hippo pathway.

**Figure 6:**
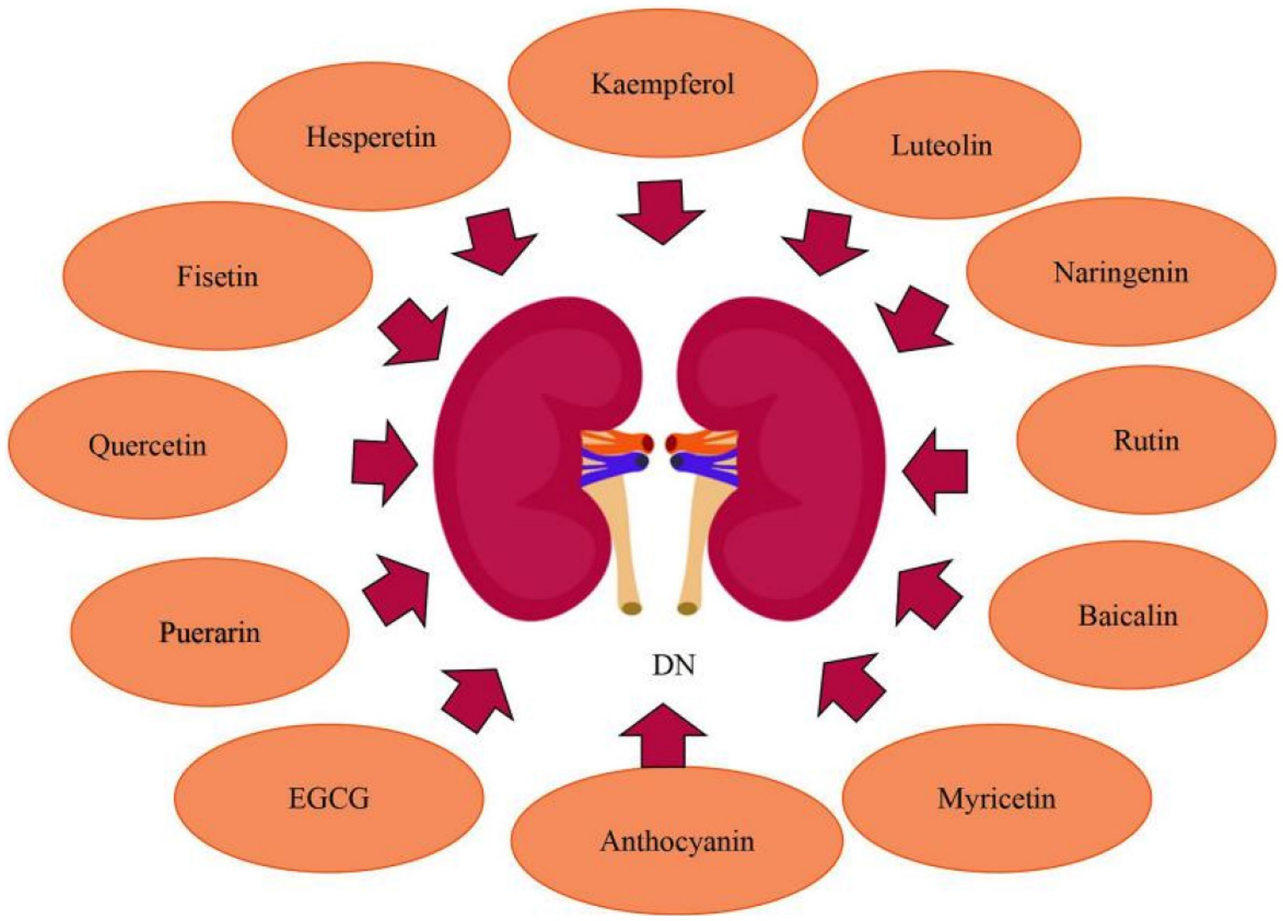
Flavonoids for the treatment of DN.

In recent years, research have discovered that EGCG, naringenin, myricetin, and fisetin can also ameliorate DN. EGCG reduces renal damage *via* modulating the degree of methylation in the Klotho gene promoter. Yang et al. (85) found that in (HG)-induced injury in HK-2 cells and diabetic db/db mice, HG treatment increased Klotho gene promoter methylation by regulating the expression of DNMT3a, whereas EGCG exerted renoprotective effects *via* its demethylation function as well as reduced Klotho gene promoter methylation in HG conditions through preventing the expression of inflammatory, fibrosis, and oxidative stress markers. Furthermore, in Klotho KO HK-2 cells treated with EGCG under HG circumstances, the expression of inflammatory, fibrosis, and oxidative stress markers was enhanced, indicating that Klotho is a primary target of EGCG *via* which it exerts its protective properties. Naringenin, a bioactive favonoid, has the potential to be used in the protection of renal. Naringenin inhibited endoplasmic reticulum stress in hyperglycemic renal cells and STZ/nicotinamide-induced diabetic rats. Naringenin administration reduced the expression of endoplasmic reticulum stress marker proteins such as ATF4, CHOP and XBP1s. Naringenin treatment inhibited the nuclear translocation of proteotoxic apoptotic marker proteins such as ATF4 and CHOP. Naringenin protected kidney damage, tubular cell apoptosis, and endoplasmic reticulum ultrastructural alterations during hyperglycemic nephrotoxicity. Besides, naringenin the management decreased the expression of TRB3, an endoplasmic reticulum stress-inducible pseudokinase. TRB3 gene silencing increased FoxO1 and Akt phosphorylation while decreasing FoxO1-mediated apoptosis during hyperglycemic nephrotoxicity, according to Khan et al. (86, 87). Given its dual target effects on oxidative stress and inflammatory pathways, myricetin may show promise in the treatment of kidney damage and dysfunction caused by diabetes. Myricetin was shown by Yang et al. (88) to decrease kidney fibrosis. Activation of the TGF-β/Smad pathway and fibrosis were induced by both oxidative stress and inflammation. Myricetin reduced oxidative stress by raising Nrf2 and decreased inflammation by blocking the IκBα/NF-κB pathway to prevent kidney fibrosis and kidney injuries. Studies have shown that fisetin improves kidney ailments. Dong et al. (89) revealed that fisetin protected mice from STZ-induced and (HG)-induced podocyte damage. Compared to healthy people, patients with DN had a reduced amount of CDKN1B mRNA expression in their glomeruli. Fisetin restored the expression of CDKN1B at the mRNA and protein levels in podocytes and mice kidney tissues. Furthermore, fisetin inhibited the phosphorylation of P70S6K, a downstream target of CDKN1B, promoted the formation of autophagosomes, and suppressed NLRP3 inflammasomes. Ge et al. (90) investigated that chronic high fat diet feeding caused considerable body weight increase in mice compared to the normal control group, as well as glucose intolerance and insulin resistance, while fisetin therapy had a significant protective effect. In the kidneys of HFD-fed mice, fisetin therapy can also decrease the expression of kidney injury molecule-1 (KIM-1) and increase the expression of nephrin and podocin, alleviating their renal dysfunction. Moeover, fisetin administration markedly restrained the inflammatory response in the kidneys of HFD-challenged mice (Figure 6).

### 3.4. Flavonoids regulate diabetes retinopathy

Diabetic retinopathy (DR) is the most common microvascular retinal complication of diabetes. Nowadays, diabetic retinopathy (DR) is classified into three types: non-proliferative diabetic retinopathy (NPDR), proliferative diabetic retinopathy (PDR), and diabetic macular edema (DME). Diabetes can disrupt the balance between inflammatory mediators and pro-survival neurotrophic factors, resulting in an inflammatory response of nerve cells and retinal endothelial cells, the production of vascular endothelial growth factor (VEGF), and the recruitment of inflammatory mediators, resulting in apoptosis of nerve cells and vascular endothelial cells and, ultimately, retinal vascular damage. AGEs caused by hyperglycemia can enhance retinal perictye loss and vascular endothelial damage (91). Hyperglycemia-induced oxidative stress results in ROS overproduction. Excessive accumulation of ROS causes apoptosis, mitochondrial damage, lipid peroxidation, inflammation, and functional and structural changes in the retina (92). Hyperglycemia causes non-enzymatic glycation of proteins, which results in the development of AGE, which may contribute to vascular endothelial damage, retinal perictye loss, and microaneurysm formation. Diabetes can cause an increase in the intraocular rennin-angiotensin system, and angiotensin II may enhance the expression of Vascular endothelial growth factor (VEGF) in retinal vascular endothelial cells. Diabetes can cause a maladaptive chronic inflammatory response in neural cells and retinal endothelial, the production of VEGF and the recruitment of inflammatory mediators, resulting in neovascularisation, apoptosis of neural cells, apoptosis of endothelial cells, and increased vascular permeability. Certain cytokines and enzymes, such as erythropoietin, VEGF, growth hormone and insulin growth factor, carbonic anhydrase, and protein kinase C, can also induce DR. If DR is not addressed, it can harm the patient’s eyesight or possibly lead to blindness, which can greatly disrupt the patient’s normal life, hence DR prevention and therapy are critical. Medication, laser photocoagulation, and surgery are now the most prevalent treatments for DR. (91) Moreover, certain flavonoids can prevent DR by suppressing endoplasmic reticulum stress, reducing oxidative stress, and up-regulating microRNA-145, etc.

**Figure 7:**
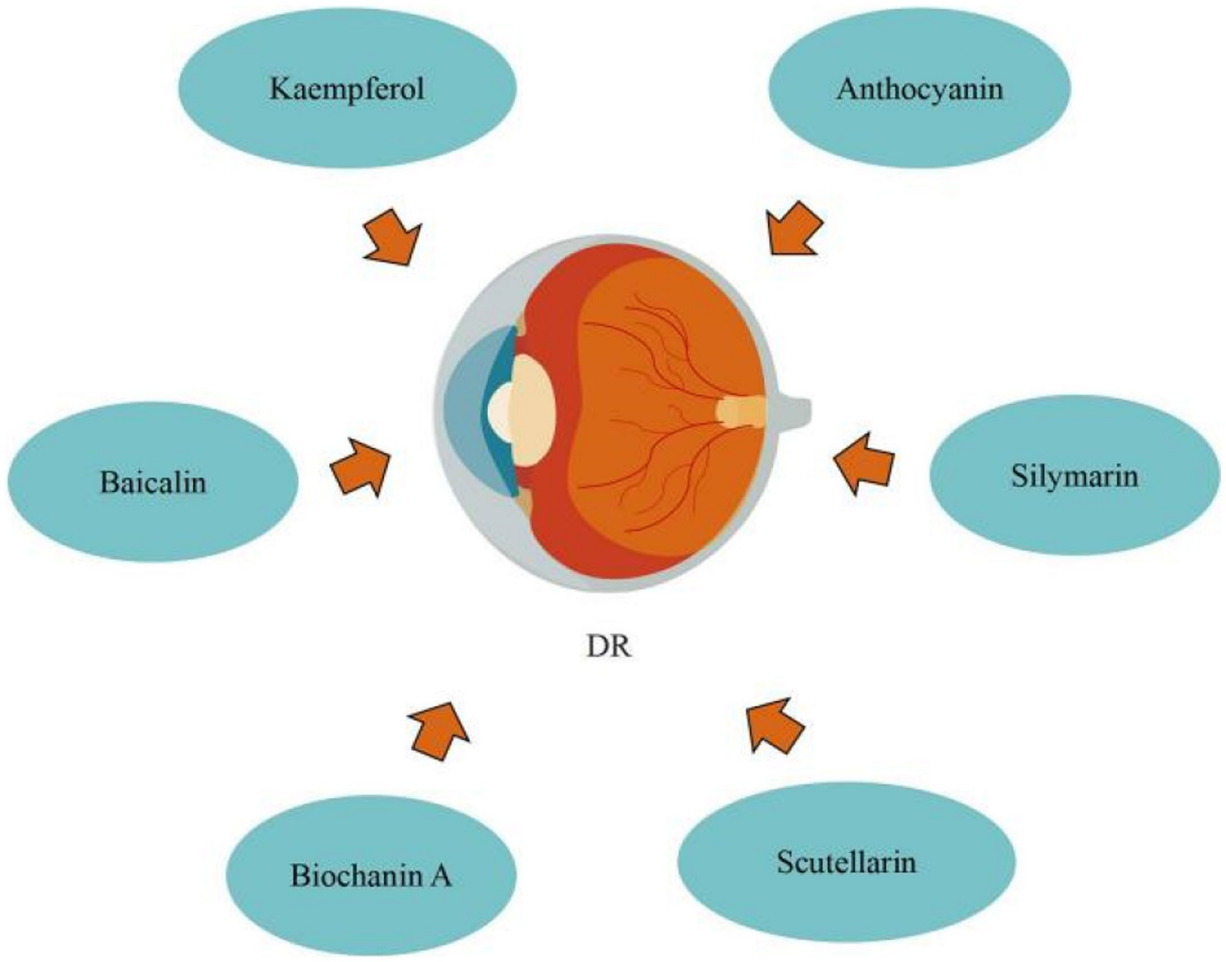
Flavonoids for the treatment of DR.

Blueberry anthocyanin extract (BAE) effectively slowed the development of DR through a molecular regulating function between ROS/reticulum stress and miR-182/OGG1 axis. Wang et al. (93) initially defined the regulatory connection between BAE and miRNA, showing that miR-182 restricts OGG1 expression directly. They also proposed that in retinal pigment epithelial cells, BAE suppressed oxidative stress and endoplasmic reticulum stress *via* the miR-182/OGG1 axis to reduce (HG)-induced apoptosis. By upregulating microRNA-145, baicalin protects human retinal microvascular endothelial cells (HRMECs) lines from HG-induced cell damage. Dai et al. (94) revealed that baicalin treatment the protective effect against HG-induced HRMECs and ARPE-19 cells injury through inhibiting p38MAPK and NF-κB signaling pathways *via* up-regulation of miR-145. Silymarin protects human retinal cells (HRECs) against diabetes-induced hyperpermeability. Garca-Ramrez et al. (95) discovered that silymarin can inhibit VEGF-induced vascular hyperpermeability of HRECs, which comprise the inner blood retinal barrier (BRB), effectively preventing BRB damage and thus reducing vascular leakage. Through modulating vasohibin-1 (VASH1), kaempferol prevents retinal ganglion cells (RGC) from (HG)-induced damage. Zhao et al. (96) discovered that kaempferol increased the expression of the protein vasohibin-1 and protected RGC against high glucose damage. Additionally, regulating ERK1/2 phosphorylation resulted in kaempferol regulation of VASH1, indicating that kaempferol may aid in the treatment of DR. By preventing retinal inflammatory responses and the oxidative stress damage that follows, which are triggered by microglia cells stimulated by hyperglycemia during DR development, scutellarin reduces BRB breakdown. According to Mei et al. (97), scutellarin prevented BRB deterioration while DR was developing. By blocking the ERK1/2-NFκB inflammatory signaling pathway, scutellarin decreased the production of the pro-inflammatory cytokine TNFα in microglia cells. Scutellarin reduced oxidative stress injury by increasing Nrf2 activation, which decreased TNFα-induced BRB damage. In addition, Scutellarin effectively and specifically suppresses DR neovascularization by suppressing the production of VEGF and activating the angiogenesis crosstalk involving p-Src, p-FAK, and p-ERK, as demonstrated by Long et al. (98) *in vitro* and *in vivo*. Significantly, in the DR rat model, scutellarin oral administration had antiangiogenic effects, indicating that oral administration could someday replace intravitreal injection in the prevention and treatment of DR. DR can be improved by biochanin A, an isoflavone found in alfalfa, cabbage, and red clover. Mehrabadi et al. (99) reported that biochanin A administration can ameliorate retinopathy via decreasing blood sugar, regulating inflammation through reducing interleukin-1beta and tumor necrosis factor-alpha, and inhibiting angiogenesis by inhibiting vascular endothelial growth factor in retinal tissues (Figure 7).

In summary, flavonoids can prevent DR by inhibiting endoplasmic reticulum stress, anti-inflammatory response, anti-oxidative stress, inhibiting VEGF pathway, upregulating microRNA-145, protecting HRECs, modulating VASH1, and suppresses DR neovascularization. Nonetheless, the absence of pharmacokinetics data is a drawback of certain research; nonetheless, medicine development studies including medicine complexes with enhanced medicine delivery methods are recommended to improve pharmacokinetics and effectiveness of flavonoids.

### 3.5. Flavonoids ameliorate other complications of T2DM

The topical administration of quercetin can promote diabetic wound recovery and regrowth. Kant et al. (100) clearly observed that treatment with quercetin raised the expression of TGF-b1, IL-10, and VEGF, while decreasing the expression of MMP-9, TNF-α, and IL-1b. Modulation of these growth factors, proteases, and cytokines resulted in formation of high-quality granular tissue, inhibition of inflammatory cells, improved angiogenesis, axonal regeneration, fibroblast proliferation, thick collagen fiber synthesis, and accelerated epithelial layer regeneration, all of which drastically enhanced diabetic wound repair and regeneration. Kaempferol has antioxidant and antiinflammatory properties, and it has been shown to heal diabetic excisional wounds. Ozay et al. (101) demonstrated that kaempferol can heal diabetic excisional wounds via growing the quantity of collagen and hydroxyproline in the wound, enhancing wound resistance, aiding wound closure, and hastening wound reepithelialization. Anthocyanin, as a nutrient, can be used to treat diabetic osteoporosis. Qi et al. (102) discovered that anthocyanin-rich black rice extract (AEBR) reduced diabetic through hindering bone marrow adipogenesis, bone turnover, as well as increasing RUNX 2 expression and the OPG/RANKL ratio. Quercetin may be implicated in endoplasmic reticulum stress in diabetic testicular secretion regulation. In diabetic rats, quercetin restricts endoplasmic reticulum stress as well as testosterone secretion disorder via the miR-1,306-5p/HSD17B7 axis, found by Wang et al. (103). Hesperetin could improve testicular damage of diabetic. Samie et al. (104) demonstrated that hesperetin could relieve diabetic testicular damage mostly by preventing apoptosis, inflammation, and oxidative stress, as well as up-regulating non-enzymatic antioxidants and endogenous enzymatic.

## 4. Conclusion and prospect

This manuscript systematically reviewed various prevalent flavonoids with hypoglycemic effect, and discussed the potential benefits of these flavonoids in T2DM and its complications. Flavonoids, as natural products, have been shown to effectively and safely ameliorate T2DM and its complications by regulating insulin resistance, glycolipid metabolism, gluconeogenesis, inflammatory response, oxidative stress, and endoplasmic reticulum stress. Therefore, exploring new flavonoids hypoglycemic medications has potential application value. However, many experiments are still remained in animals and *in vitro*, so it is necessary to investigate the specific mechanism of flavonoids affecting diabetes at a deeper level from the molecular level, in order to provide an important theoretical basis for its clinical promotion and to provide fresh ideas for the prevention and treatment of diabetes.

## Author contributions

XY and MD jointly contributed to the topic selection and writing of the paper. YY and YS contributed to the guidance of this paper. All authors contributed to the article and approved the submitted version.

## Funding

This work was supported by research grants from 2023 Liaoning Province Applied Basic Research Project (20230319).

## Conflict of interest

SW was employed by Liaoning Shengqi Haotian Biomedical Technology Co., Ltd.

The remaining authors declare that the research was conducted in the absence of any commercial or financial relationships that could be construed as a potential conflict of interest.

## Publisher’s note

All claims expressed in this article are solely those of the authors and do not necessarily represent those of their affiliated organizations, or those of the publisher, the editors and the reviewers. Any product that may be evaluated in this article, or claim that may be made by its manufacturer, is not guaranteed or endorsed by the publisher.
